# Supplementary material for: Reversal of Postnatal Brain Astrocytes and Ependymal Cells towards a Progenitor Phenotype in Culture
Source: Cells. 2024 Apr 12;13(8):668. doi: 10.3390/cells13080668 (PMC11049274; doi:10.3390/cells13080668)
Supplement: Supplementary file 1 [file cells-13-00668-s001.zip › cells-2776102-supplementary.pdf]

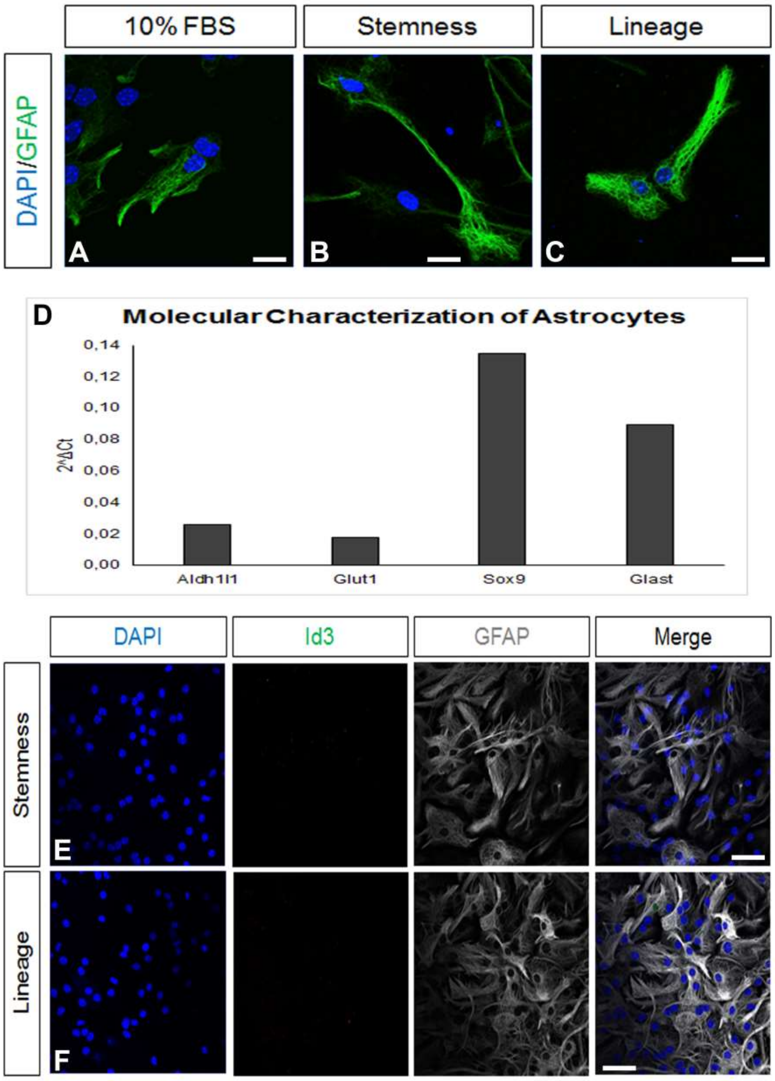

Sup. Figure S1. Morphologic and marker profile comparison of typical and de-differentiated astrocytes

(A-C) Images of cells immunostained for GFAP (in green) after culture for 7 days in DMEM+ 10%FBS, or after a further 7-day culture in the two different de-differentiation media. (D)Graph showing the gene expression (using reverse, real-time PCR) of the cells cultured in 10% FBS for 7 days, indicating a mature astrocyte profile. (E-F) Images of cells immunostained for GFAP (in grey) and for ID3 (in green) after culture in the two different de-differentiation media. [scale bars in A-C: 5μm and in E-F: 15μm]

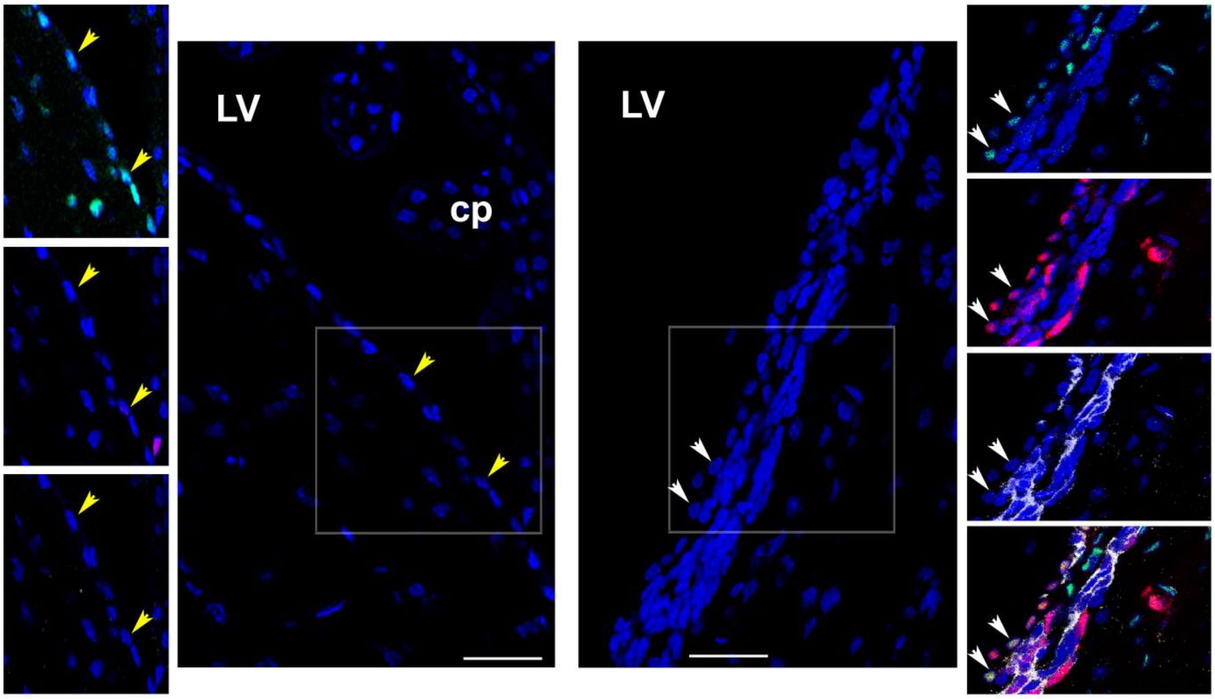

Supp. Figure S2. Appearance of proliferating ependymal cells after brain milking

High magnification images the SEZ niche at 3 months post-milking, in rat brain sections immunostained for Sox2 (in green) to mark ependymal cells, PCNA (in red) to mark proliferating cells and Dcx (in grey) to mark neuroblasts. At the left is shown the medial ventricular wall, which is not neurogenic. Note the absence of Dcx+ cells and that the ependymal cell layer is formed by Sox2-expressing cells (yellow arrowheads indicate two of these). At the right side is shown the lateral wall of the ventricle, where the SEZ is located. Note the presence of neuroblasts, of the Sox2+ ependymal layer, where PCNA is also present (as indicated by white arrowheads). [Scale bars: 30μm]
